# MicroRNA-187 inhibits tumor growth and invasion by directly targeting CD276 in colorectal cancer

**DOI:** 10.18632/oncotarget.10023

**Published:** 2016-06-14

**Authors:** Zheng-Shi Wang, Ming Zhong, Yu-Hai Bian, Yi-Fei Mu, Shao-Lan Qin, Min-Hao Yu, Jun Qin

**Affiliations:** ^1^ Department of Gastrointestinal Surgery, Ren Ji Hospital, School of Medicine, Shanghai Jiao Tong University, Shanghai 200127, P.R. China

**Keywords:** microRNA-187, CD276, colorectal cancer, growth, invasion

## Abstract

Aberrantly expressed microRNAs contribute to the initiation and progression of human cancers. However, the underlying functions of microRNA-187 (miR-187) in colorectal cancer (CRC) remain largely unexplored. Here, we demonstrated that miR-187 was significantly down-regulated in CRC tissues and cell lines compared to their normal counterparts. By Kaplan-Meier analysis, we revealed that decreased miR-187 expression was closely associated with shorter overall survival and relapse-free survival of patients with CRC. By gain- and loss-of-function studies, we showed that miR-187 remarkably suppressed CRC cell proliferation, migration, invasion, and promoted cell apoptosis. Furthermore, bioinformatics analysis and luciferase reporter assay identified that CD276 was the direct functional target of miR-187 in CRC. Genetic silencing of CD276 recapitulated similar phenotype as observed in over-expression of miR-187, and restoration of CD276 completely rescued the inhibitory effect of miR-187 in CRC cells. Taken together, our study implied the essential roles of miR-187 in suppressing CRC progression, and a novel link between miR-187 and CD276 in CRC.

## INTRODUCTION

Colorectal cancer (CRC) is the third leading cause of cancer-related deaths worldwide [1], resulting in more than 600,000 deaths each year. However, promising therapy for CRC is not available, for the molecular mechanisms of CRC development and progression remain ambiguous. Thus, better understanding the pathogenesis of CRC and exploring novel targets is of crucial significance for CRC treatment [2].

MicroRNAs (miRNAs) are a class of small (approximately 20-22 nucleotides) endogenous noncoding RNA molecules [3]. It mediates negative posttranscriptional regulation by base pairing with the 3′ UTRs of one or more target genes [4]. Accumulating evidence has demonstrated that aberrant expression of miRNAs act as tumor suppressors or oncogenes in various human cancers, including CRC, breast cancer, lung cancer and prostate cancer [5–8]. Recent study showed that miR-187 is significantly down-regulated in many types of human malignancies. For example, miR-187 in breast cancer leads to a more aggressive, invasive phenotype and acts as an independent predictor of outcome [9]. In ovarian cancer, miR-187 regulates tumor progression through targeting Disabled homolog-2 (Dab2), which resulted in inhibition of epithelial-mesenchymal transition [10]. In pancreatic cancer, miR-187 can predict short overall survival (OS) in patients after radical surgery [11]. However, its possible functions and underlying mechanisms in CRC have not been reported yet.

CD276, as an immunoregulatory molecule, plays immunological and non-immunological roles in different types of human cancer including CRC [12]. The precise functions of CD276 in tumor immunity are complicated as both T cell co-stimulatory and co-inhibitory effects have been reported [13]. Recently, emerging studies reveal that high tumor CD276 expression is correlated with more advanced disease and poor prognosis [9, 14, 15]. And CD276 also affects tumor progression and chemosensitivity by regulating oncogenic signaling pathways activated in non-immunological systems [16, 17]. In colorectal cancer, nuclear CD276 expression strongly predicts poor outcome and associates with clinicopathological parameters. Overexpression of CD276 contributes to apoptosis-resistance in CRC cell lines by elevating the Jak2-STAT3 pathway [18]. However, the oncogenic activities of CD276 in CRC remain a large area to investigate.

In the current study, we revealed significant down-regulation of miR-187 in CRC tissues and cell lines. Over-expression of miR-187 suppressed CRC cell proliferation, migration and invasion, and promoted CRC cell apoptosis. Inversely, knockdown of miR-187 significantly facilitated the malignant phenotype of CRC cells. Furthermore, we identified CD276 as a direct and functional target of miR-187. Therefore, our results suggest that miR-187 may play crucial roles in the development and progression of CRCs by targeting CD276.

## RESULTS

### MiR-187 is down-regulated in CRC and correlated with prognosis of CRC patients

**Figure 1:**
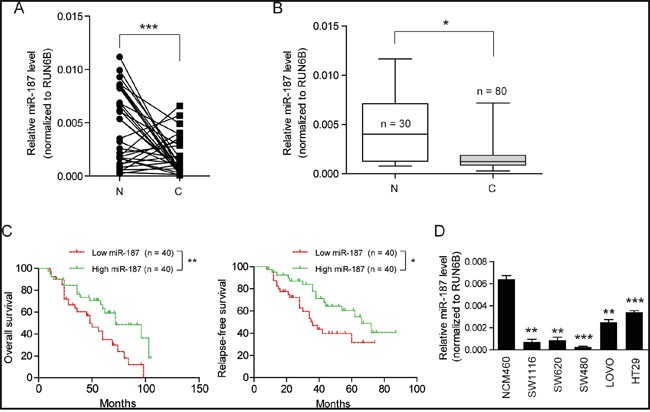
MiR-187 is down-regulated in CRC and correlated with prognosis of CRC patients **A.** The transcription level of miR-187 in 32 matched CRC tissues (T) and adjacent normal tissues (N) by quantitative real-time PCR and normalized by an endogenous control, U6 RNA, ***P < 0.001. **B.** The mRNA expression level of miR-187 in 80 tumor tissues (T) and 30 non-tumor tissue (N) was detected by Real-time quantitative PCR, *P < 0.05. **C.** Kaplan-Meier curves for patients grouped based on miR-187 expression; *P < 0.05; **P < 0.01. **D.** Histograms of the transcription level of miR-187 in human CRC cell lines SW1116, SW480, SW620, HT29 and LOVO, and the normal colonic epithelial cells NCM460; **P < 0.01; **P < 0.001.

To elucidate the expression pattern of miR-187 in CRC, we first detected the expression level of miR-187 in 32 matched CRC tumor and non-tumor tissues by Real-time PCR. The result showed that miR-187 was reduced in CRC tissues compared with matched cancer-adjacent tissues (Figure 1A). To further confirm this observation, miR-187 expression in another cohort containing 30 normal colorectal tissues and 80 CRC tissues with follow-ups was analyzed. As shown in Figure 1B, miR-187 was significantly decreased in CRC specimens in relative to the normal control. Meanwhile, the prognostic value of miR-187 expression was also analyzed in CRC patients. Kaplan-Meier curve revealed that the overall survival rate and relapse-free survival rate of the low miR-187 expression group was higher than those of the high miR-187 expression group (Figure 1C). Subsequently, we measured the expression levels of miR-187 in 5 CRC cell lines and the normal colonic epithelial cell line NCM460 by Real-time RT-PCR (Figure 1D). As showed, miR-187 expression was relatively low in 3 cell lines (SW1116, SW480 and SW620), while that was relatively high in the other 2 cell lines (LOVO and HT29). Notably, the highest miR-187 level was detected in NCM460 cells. Collectively, these findings indicate that down-regulatedmiR-187 correlates CRC patients’ clinical outcome and might play a role in CRC development or progression.

### MiR-187 inhibits cell proliferation, migration, invasion and promotes apoptosis in CRC cells

**Figure 2:**
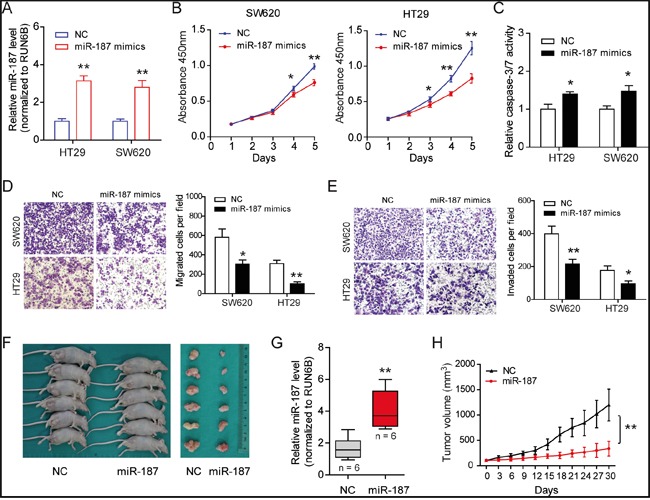
MiR-187 inhibits cell proliferation, migration, invasion and promotes apoptosis in CRC cells **A.** The expression level of miR-187 was detected after transfection of mimics. **B.** Over-expression of miR-187 inhibited proliferation in SW620 and HT29 cells detected by CCK-8 assays. **C.** MiR-187 increased the caspase-3/7 activity of SW620 and HT29 cells compared with miR-187 mimics-NC-transfected cells (*P < 0.05). Transwell assay was performed to detect the migratory **D**. and invasion **E**. potential. Over-expression of miR-187 significantly decreased the invasive potential of SW620 and HT29 cells when transfected with miR-187 mimics compared with control cells (*P < 0.05, **P < 0.01). **F.** Subcutaneous tumor transplantation experiment was performed. **G.** The miR-187 level was detected in tumor tissues upon miR-187 Agomir and Agomir NC treatment (**P < 0.01). **H.** The mass of the tumors in the group with exogenous miR-187 Agomir was lower than that in the group with exogenous miR-187 Agomir NC, **P < 0.01.

In order to investigate the function of miR-187 in CRC, we transfected transiently miR-187 mimics into SW620 and HT29 cells and measured cellular functions (Figure 2A). Using CCK-8 assays, we observed that the growth rate of miR-187 mimics treated cells was inhibited compared with NC mimics-transfected cells (Figure 2B). Consistently, elevated caspase-3/7 activity was also observed in miR-187 mimics group (Figure 2C). To determine whether miR-187 can affect CRC cell invasive capacity, we performed Transwell assay. The result showed that miR-187 significantly decreased the migratory (Figure 2D) and invasive (Figure 2E) potential of SW620 and HT29 cells. To further confirm the role of miR-187 on tumor growth, we performed subcutaneous tumor transplantation experiment (Figure 2F). Exogenous miR-187 Agomir increased the level of miR-187 in tumor microenvironment and inhibited the growth of SW620 cells in nude mice (Figure 2F and 2G). Then the tumors were excised and measured, the mass of tumors in the group with exogenous miR-187 Agomir was lower than that in the group with exogenous miR-187 Agomir NC (Figure 2H). Taken together, these results suggest that miR-187 inhibits tumor growth of CRC *in vitro* and *in vivo*.

### Knockdown of miR-187 promotes cell proliferation, invasion and inhibits apoptosis in CRC cells

**Figure 3:**
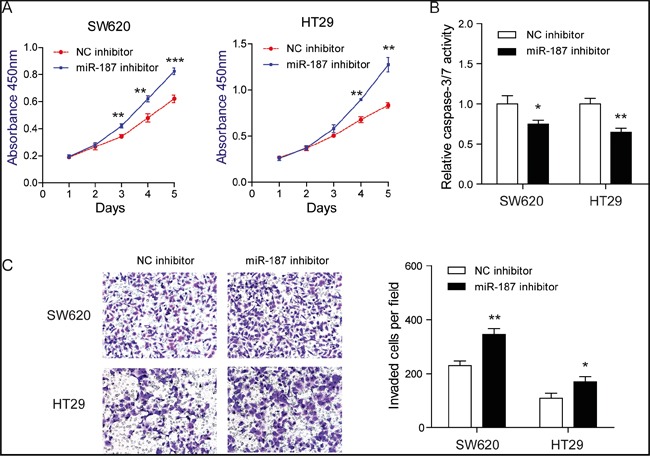
Knockdown of miR-187 promotes cell proliferation, invasion and inhibits apoptosis in CRC cells **A.** Inhibition of miR-187 promoted proliferation in SW620 and HT29 cells detected by CCK-8 assays (**P < 0.01, ***P < 0.001). **B.** Inhibition of miR-187 decreased the caspase-3/7 activity of SW620 and HT29 cells (*P < 0.05, **P < 0.01). **C.** Inhibition of miR-187 with miR-187 inhibitor significantly decreased the invasive potential by Transwell assay (*P < 0.05, **P < 0.01).

To demonstrate whether endogenous miR-187 contributes to tumor progression, we transfected SW620 and HT29 cells with miR-187 inhibitor or miR inhibitor control. Expectedly, suppression of miR-187 significantly promoted cell proliferation (Figure 3A) and invasion (Figure 3C) of CRC cells. Besides, starvation-induced cell apoptosis in SW620 and HT29 cells was inhibited (Figure 3B). Taken together, these observations indicate that miR-187 inhibits tumor progression of CRC by negatively controlling these cellular phenotypes.

### CD276 is a direct target of miR-187

**Figure 4:**
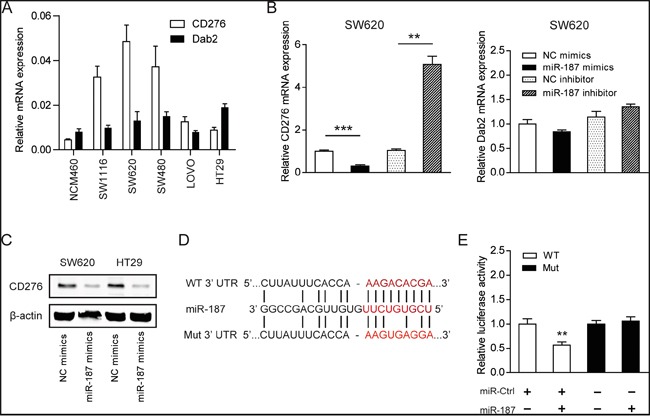
CD276 is a direct target of miR-187 **A**. Expression level of CD276 and Dab2 in CRC cell lines and the normal NCM460 cells. **B.** The mRNA level of CD276 and Dab2 were detected in the presence of miR-187 mimics or miR-187 inhibitor (**P < 0.01, ***P < 0.001). **C.** The protein level of CD276 in SW620 and HT29 cells was detected in the presence of miR-187 mimics by Western blotting. **D.** The predicted sites of miR-187 binding to the 3′-UTR region of CD276 were detected using bioinformatics prediction tools. The mutated site in the 3′-UTR region of CD276 was also shown. **E.** SW620 cells were co-transfected with miR-187 expression vector or the empty vector and CD276 3′-UTR reporter plasmid or its mutant form. And the luciferase activity was detected 48 h after transfection. The data are shown as the means ± S.D. of three replicates (**P < 0.01).

To explore the mechanism by which miR-187 affects the biological functions of CRC cells, we next aimed to investigate the potential gene targets of miR-187 using target prediction programs including, MIRDB and DIANA-MICROT. Our analysis revealed that CD276 and Dab2 are two potential targets of miR-187. CD276 and Dab2 were commonly up-regulated in CRC cells compared to the normal NCM460 cells (Figure 4A). Interesting, CD276, but not Dab2, was negatively correlated miR-187 level in CRC cells (Figure 1D and 4A), indicating CD276 might be the target of miR-187 in CRC. To certify this prediction, we detected the mRNA expression of CD276 and Dab2 in SW620 cells in the presence of miR-187 mimics or miR-187 inhibitor. The resulted showed that CD276 mRNA expression was significantly reduced by treatment of miR-187 mimics, and remarkably increased by treatment of miR-187 inhibitor, while the mRNA expression of Dab2 was faintly influenced by these stimulation or inhibition (Figure 4B). As demonstrated by Western blotting, miR-187 decreased the levels of CD276 protein in SW620 and HT29 cells (Figure 4C), which supports our hypothesis to a certain extent.

To verify whether or not that CD276 is a direct target of miR-187, then, a human CD276 3′UTR fragment containing the wild-type or mutant miR-187-binding site was inserted downstream of the luciferase open reading frame (Figure 4D). These reporter constructs were used to co-transfect miR-187 mimics cells or control cells. As shown in Figure 4E, the relative luciferase activity of the reporter containing wild-type CD276 3′-UTR was markedly decreased upon miR-187 co-transfection, whereas the luciferase activity of the reporter containing the mutant binding site was unaffected. These results strongly suggest that CD276 is a direct target of miR-187 in SW620 cells.

### Silencing of CD276 compromises the tumor progression of CRC *in vitro*

**Figure 5:**
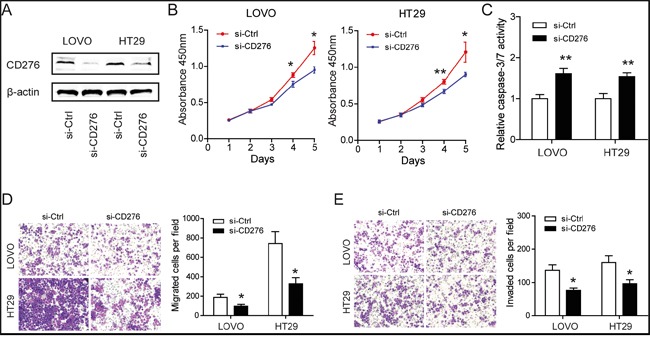
Silencing of CD276 compromises the tumor progression of CRC *in vitro* **A.** Efficacy of RNA interference in LOVO and HT29 cells was verified by Western blotting. **B.** Silencing of CD276 inhibited proliferation in LOVO and HT29 cells detected by CCK-8 assays (*P < 0.05, **P < 0.01). **C.** Silencing of CD276 increased the caspase-3/7 activity of LOVO and HT29 cells compared with siRNA control transfected cells (**P < 0.01). Inhibition of CD276 significantly decreased the migratory **D**. and invasion **E**. ability of LOVO and HT29 cells (*P < 0.05). Data represent means ± S.D. of at least three independent experiments.

To reveal the role of CD276 in CRC, specific siRNAs against CD276 were designed and synthesized. The interference efficacy was measured by Western blotting. As shown in Figure 5A, CD276 protein level was markedly reduced upon siRNAs treatment. Consistent with the functions of CD276 in CRC [19] and hepatocellular carcinoma [14, 20], knockdown of CD276 resulted in growth arrest as evidenced by reduced cell viability (Figure 5B) and increased cell apoptosis (Figure 5C). Meanwhile, similar to the phenotype induced by treatment of miR-187 mimics, silencing of CD276 also decreased migratory ability and invasive potential of LOVO and HT29 cells (Figure 5D and 5E).

### Restoration of CD276 abolishes the tumor suppressor role of miR-187

**Figure 6:**
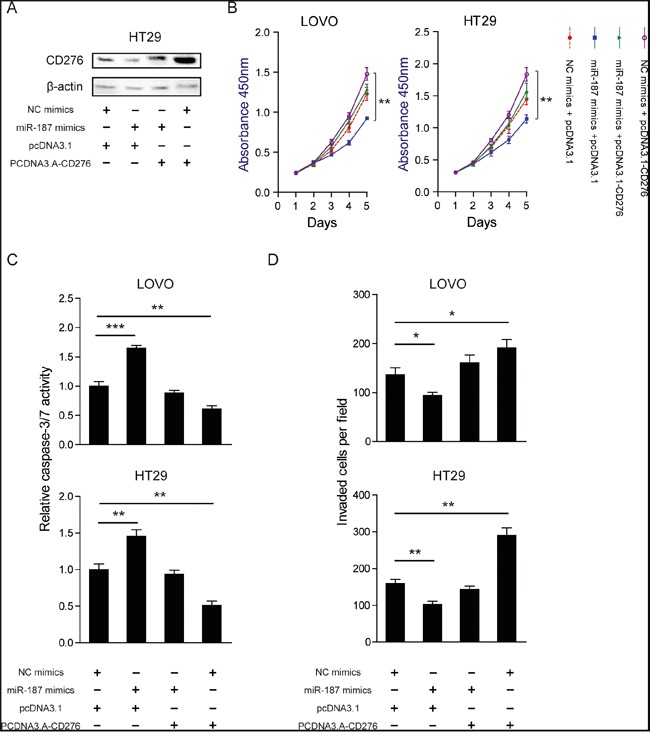
Restoration of CD276 abolishes the tumor suppressor role of miR-187 LOVO and HT29 cells were transfected with NC mimics or miR-187 mimics together with either pcDNA3.1 or pcDNA3.1-CD276. **A.** The protein level of CD276 was measured in those indicated treatment in HT29 cells. CCK-8 assay **B**., caspase-3/7 activity **C**. and Transwell assay **D**. were performed to examine cell proliferation, apoptosis, and invasion abilities of the transfected cells, respectively. The data are shown as the means ± S.D. *P < 0.05, **P < 0.01, ***P < 0.001.

To further confirm whether the tumor-suppressive roles of miR-187 were mediated by CD276, a gain-of-function study was performed. A vector expressing CD276 without its 3′UTR was constructed in LOVO and HT29 cells. The protein level of CD276 was detected in HT29 cell (Figure 6A). As shown in Figure 6B-6D, over-expression of CD276 completely abolished the effects of miR-187 on cell proliferation, apoptosis and invasion in LOVO and HT29 cells. Collectively, these data strongly indicate that CD276 is the direct functional mediator of miR-187.

## DISCUSSION

Few advances in the treatment for CRC have been made over the past decade. The discovery of miRNAs provides a novel insight into understanding the molecular mechanisms and curing for cancers. The roles of miRNAs in various cancers have been attracting more and more attention. In this study, we first measured the expression of miR-187 in CRC tissues and cell lines, and found it was significantly down-regulated compared with corresponding normal controls. Biological functions of miR-187 in CRCs were investigated using gain- and loss-of-function studies. Previously, down-regulation of miR-187 predicts a poor prognosis has been reported in clear cell renal cell carcinoma (ccRCC), which demonstrated that down-regulated miR-187 is associated with higher tumor grade and stage, and plays a tumor-suppressive role [21]. However, increased expression of miR-187 in ovarian cancer correlated with the better prognostic group, and showed distinct dual roles in cell proliferation and tumor progression of ovarian cancer [10]. These findings suggest the expression pattern functions of miR-187 may be tissue specific. Consistent with the observation in ccRCC, we also demonstrated miR-187 acts as a tumor suppressor in CRC in cell proliferation, apoptosis and invasive capacity. In addition to *in vitro* experiment, we validated our conclusion in the nude mice. In this experiment, the nude mice were treated with miR-187 Agomir, which is similar to miR-187 mimics but more stable *in vivo* [22]. The tumor sizes in the experiment group were significantly smaller than that in the control group, which was consistent with the *in vitro* result. However, further research is still needed to explore whether other miRNAs can regulate CD276 since miR-29a can also directly target CD276 molecule [23].

In order to explore the mechanisms of miR-187, we identified CD276 as a putative miR-187 target gene. CD276, also known as B7-H3, an immunoregulatory protein that belongs to the B7 family of T-cell co-regulatory molecules [24], has either stimulatory or inhibitory immunological effects [25–27]. In CRCs, the prognostic value of CD276 was studied by many groups. The results suggested that CD276 expression correlated with more advanced pathological grade and associated with reduced metastasis-free, disease-specific and overall survival; and these findings suggest that CD276 might be involved in CRC progression and metastasis [28, 29]. Furthermore, it has been reported that CD276 participated in the process of miRNA-related regulation [23]. Therefore, we selected CD276 for further study. Indeed, we confirmed that CD276 mRNA and protein were down-regulated by the ectopic expression of miR-187, as showed by Real-time-PCR and Western blotting, respectively. Also we proved that CD276 is a direct target of miR-187 by dual luciferase reporter gene assay. And restoration of CD276 completely abrogated the tumor suppressor role of miR-187. The oncogenic activities of CD276 have been reported in many human cancers, including acute monocytic leukemia [30], non-small cell lung cancer [31], gastrointestinal carcinoma [15, 20, 32], and mantle cell lymphoma [33]. Despite the majority of studies on CD276 and cancer emphasize the paradox immunological role of CD276, several studies showed that CD276 can regulate tumor progression and chemosensitivity by targeting with signaling pathways involved in non-immunological systems. However, the precise molecular mechanisms for the functional role of CD276 in cancer remain elusive. In melanoma and breast cancer, CD276 has been reported to promote metastasis and chemoresistance through regulating JAK2/STAT3 signaling pathways and promoting the expression of cytokines and other metastasis-associated genes [16, 17]. The positive regulation of JAK2/STAT3 signaling pathways has also implicated in CD276-mediated epithelial-to-mesenchymal transition in liver cancer [20]. However, whether JAK2/STAT3 signaling pathways is involved in the oncogenic activities of CD276 in CRC remain further investigation.

In summary, the present study provides the essential roles of miR-187 in negatively regulating CRC progression and a novel link between miR-187 and CD276 in CRC. Potent monoclonal antibody of CD276 is already available and has been reported as being safe for treatment of advanced-stage central nervous system cancer in children [34]. The miR-187/CD276 axis provides novel insight into the pathogenesis of CRC, and might represent a potential therapeutic target for the treatment of CRC.

## MATERIALS AND METHODS

### Clinical samples

A total of 32 pairs of CRC tumor and matched non-tumor tissues, and 80 CRC tissues as well as 30 normal colorectal tissues were collected at the Department of General Surgery, Ren Ji Hospital, School of Medicine, Shanghai Jiao Tong University between January 2002 and February 2013. None of the patients had received neoadjuvant chemotherapy before operation. They were all verified to be patients with CRC by H&E staining after operation. Fresh CRC tissues and matched cancer-adjacent tissues were sampled directly after surgical removal, and immediately frozen in liquid nitrogen for further use. The follow-up time was calculated from the date of surgery to the date of death, or the last known follow-up. All patients were well informed and the process was approved by Ethics Committee of Ren Ji Hospital, School of Medicine, Shanghai Jiao Tong University, China.

### Cell culture

Human CRC cell lines SW1116, SW480, SW620, HT29, LOVO and the normal colonic epithelial cell line NCM460 were all obtained from American Type Culture Collection (ATCC, Manassas, VA, USA) and kept in our laboratory. Cells were cultured in RPMI-1640 (Gibco) supplemented with 10% fetal bovine serum (FBS) and 1% antibiotics (100 μg/ml streptomycin and 100 units/ml penicillin). Cultures were maintained at 37°C under an atmosphere containing 5% CO_2_.

### Total RNA extraction and real-time PCR

Total RNA was extracted using RNAiso Plus (TaKaRa, Biotech Co., Ltd, Dalian, China). cDNA was synthesized using M-MLV MicroRNA Reverse Transcription Kit (Promega, USA). Real-time PCR was performed with SYBR Premix Ex TaqTM (TaKaRa, Biotech Co., Ltd, Dalian, China). PCR primer for miR-187 was TCGTGTCTTGTGTTGCAGC (forward) and GTGCAGGGTCCGAGGT (reverse). The expression levels were normalized to U6. PCR primer for U6 was CTCGCTTCGGCAGCACA (forward) and AACGCTTCACGAATTTGCGT (reverse). PCR was performed under the following conditions: 94°C for 4 min, followed by 40 cycles at 94°C for 30 s, 50°C for 30 s and 72°C for 40 s. Each sample was run in triplicate. The primers for CD276 and Dab2 were shown as follows. CD276, forward 5′-ACAGGAAGATGCTTCGAGGA-3′, reverse 5′-GAGACCTGGACTTCCACAGC-3′; Dab2, forward 5′-GTAGAAACAAGTGCAACCAATGG-3′, reverse 5′-GCCTTTGAACCTTGCTAAGAGA-3′; β-actin, forward 5′-ACTCGTCATACTCCTGCT-3′, reverse 5′-GAAACTACCTTCAACTCC-3′. Relative expressions were determined by normalizing expression of each Ct value to β-actin Ct value and data were analyzed according to the 2^−ΔΔCt^ formula.

### Cell proliferation assay

A density of 3,000 indicated CRC cells/well upon different treatments were seeded in a 96-well cell culture plate, grown at 37°C overnight. The cell viability was quantified by Cell Counting Kit-8 (CCK-8, Dojindo, Japan). Briefly, on the day of measuring the growth rate of treated cells, 100 μl of spent medium was replaced with an equal volume of fresh medium containing 10 % Cell Counting Kit-8 (CCK-8), then cells continued to be incubated at 37°C for 1 h, and the absorbance was finally determined at 450 nm using a micro plate reader.

### Transwell cell migration and invasion sssay

The migratory and invasive ability of CRC cells was detected by transwell model (Corning, NY) according to the manufacturer's instructions. For cell invasion assay, 5×10^4^ indicated CRC cells were placed on the upper chamber of each insert coated with 100 μl of 2 mg/ml growth factor reduced Matrigel (BD Bioscience, USA), and 700 μl of RPMI 1640 with 5% FBS was added to the lower part of the chamber. After incubating for 60 h, the chambers were disassembled, the non-invaded cells that remained on the upper chamber were removed, and the membranes were stained with a 2% crystal violet solution for 30 min and placed on a glass slide. Then, cells that had migrated across the membrane were counted in five random visual fields using a light microscope. Specially, for cell migration assay, no Matrigel was coated and the incubation time was 24 h. All assays were performed three independent times in triplicate.

### Apoptosis assay

To perform caspase-3/7 activity assay, CRC cells were seeded on 96-well plates at a density of 10,000 cells per well. After starvation for 48 h, cell number and caspase-3/7 activity were monitored on the same sample using CellTiter-Blue (Promega, USA, #G8081) and Apo-ONE Caspase-3/7 assay (Promega, USA, #G7790), respectively. Caspase-3/7 activity was calculated as the ratio Apo-ONE/CellTiter-Blue signals. The measurement was performed in triplicate.

### miRNA targets prediction

The putative miRNA targets were predicted using the MIRDB (http://mirdb.org/cgi-bin/search.cgi) and DIANA-MICROT (http://diana.cslab.ece.ntua.gr/microT/) algorithms.

### Western blotting

Western blotting was performed to determine CD276 protein expression. All proteins were resolved on a 10% SDS-denatured polyacrylamide gel and were then transferred onto a nitrocellulose membrane. Membranes were incubated with blocking buffer for 90 min at room temperature and then incubated with an antibody against CD276 (Abcam, ab105354) or β-actin overnight at 4°C. The membranes were washed and incubated with a horseradish peroxidase (HRP)-conjugated secondary antibody. Protein expression was assessed by enhanced chemiluminescence and exposure to chemiluminescent film. The LabWorks image acquisition and analysis software (UVP, LLC) was used to quantify band intensities. All antibodies were purchased from Epitomics (California, USA).

### Plasmid construction, transfection with miRNA and small interfering RNA

The coding sequences of CD276 were cloned into pcDNA3.1 (+) to generate CD276 expression vectors. The wild-type CD276 3′UTR was cloned into the pMIR-REPORT luciferase vector (Ambion, Austin, TX, USA). Mutant CD276 3′UTR was generated based on the pMIR-CD276-3′UTR by mutating 3 nt that are recognized by miR-187. The primers for CD276 were: 5′-TGTGGATCCCTGTCATCTGGGAAGTAACAACGCA-3′ (forward) and 5′-AAGTCTAGAGAGCCACTACTGCCTGTTGTCTTTG-3′ (reverse). The primers for CD276 3′UTR were: 5′-TCTGAGCTCGCTAAACAGCCATAAACGGAAACGC-3′ (forward) and 5′-ACCACGCGTGCGTAGATTCTCCTTTATGGGGCTG-3′ (reverse). MiR-187 mimics, miR mimic control, miR-187 inhibitor, miR inhibitor control, and small interfering RNA targeting CD276 were purchased from GenePharma (Shanghai, China). Transfection was performed according to the manufacturer's instructions.

### Luciferase reporter assays

The reporter plasmid was transiently transfected into SW620 cells in the presence of either miR-187 or miR-control. After 48 h, the cells were harvested and lysed, and luciferase activity was measured using the Dual-Luciferase Reporter Assay System (Promega, Madison, WI, USA). Renilla-luciferase was used for normalization. The experiments were performed independently in triplicate.

### Tumorigenesis in nude mice

Xenograft tumors were generated by subcutaneous injection of 4 × 10^6^ cells on the hind limbs of each 4-to-6-week-old BALB/C athymic nude mouse (nu/nu) obtained from the Animal Center of East China Normal University, Shanghai, China. All mice were housed and maintained under specific pathogen-free conditions and used in accordance with institutional guidelines and approved by the Use Committee for Animal Care. Tumor size was measured by a slide caliper every 3 days and tumor volume was determined by the formula 0.44 × A × B^2^ (A indicates tumor base diameter one direction and B the corresponding perpendicular value). When the average value of tumor sizes was up to 100 mm^3^, the mice were separated into 2 groups randomly, one with subcutaneous injection of miR-187 (Agomir) at different sites, and the other with miR-187 (Agomir) NC. Injection was performed twice a week. All mice were euthanized at 30 days after the initial injection, and the tumors were excised.

### Statistical analyses

Data were expressed as the means ± SEM of at least three independent experiments. All statistical analyses were performed using the SPSS 16.0 software and graphical representations were performed with GraphPad Prism 5 (San Diego, CA) software. Overall survival rate was calculated according to the Kaplan-Meier method and the difference in survival curves was evaluated by the log-rank test. The Student's t-test was used to analyze differences between two groups, and one-way ANOVA was used to determine the significance of differences among multiple groups. P values less than 0.05 were considered statistically significant.
